# An operative case of gastric cancer with ectopic bile duct drainage in the lesser curvature of the stomach

**DOI:** 10.1186/s40792-024-01862-5

**Published:** 2024-03-21

**Authors:** Kazuaki Hashimoto, Tsukasa Ihara, Yuichiro Maruyama, Shinichi Tomisaki, Hiroshi Harada

**Affiliations:** Department of Surgery, Sasebo Kyousai Hospital, 10-17 Shimanjicho, Sasebo, Nagasaki Japan

**Keywords:** Double common bile duct, Ectopic drainage, Gastric cancer, Malformation

## Abstract

**Background:**

Among the various anomalies of the biliary system, a double common bile duct with ectopic drainage in the stomach is rare. Furthermore, ectopic bile ducts are extremely rare in gastric cancers.

**Case presentation:**

A 67-year-old man was admitted to our hospital with gastric cancer and ectopic left extrahepatic bile duct drainage in the stomach. Pre-operative testing revealed no communication between the intrahepatic bile ducts. Distal gastrectomy and bile duct jejunostomy were performed. The post-operative course was uneventful, and the patient did not exhibit recurrence for 39 mo.

**Conclusions:**

Although it is uncertain whether sustained bile exposure from an ectopic bile duct is related to gastric cancer, short-term follow-up might be necessary because of the possibility of gastric cancer.

## Introduction

Anatomical anomalies of blood vessels and bile ducts are often discovered during upper abdominal surgery [1]. However, ectopic bile duct drainage in the lesser curvature of the stomach is rare. This report presents a rare case of ectopic bile duct drainage in the stomach that possibly caused gastric cancer.

## Case presentation

**Fig. 1:**
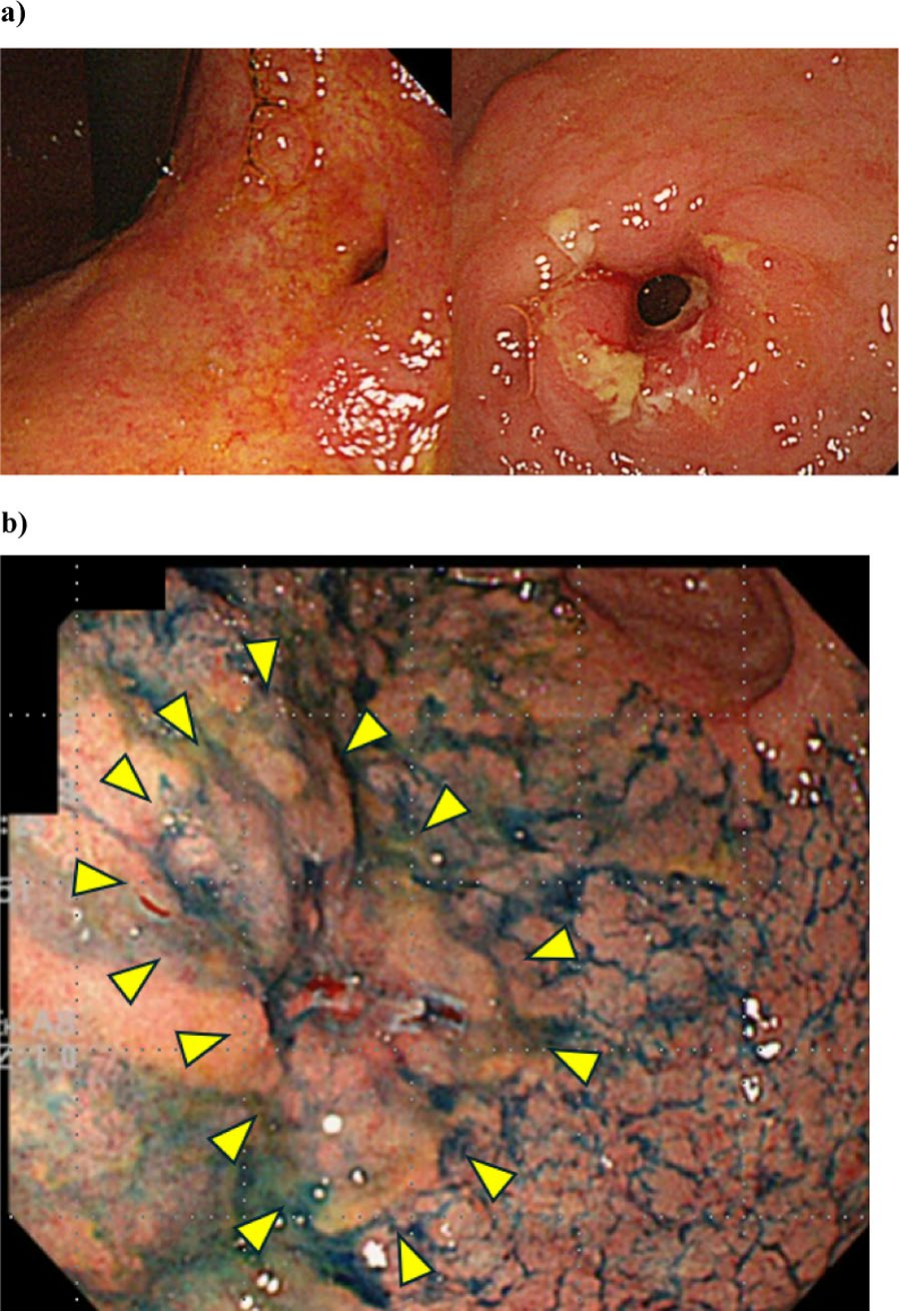
Upper gastrointestinal endoscopy. **a** Bile leakage from the foramen opening to the lesser curvature of the stomach. **b** The area enclosed by the triangle represents the suspected tumor area. Macroscopic type 0–IIa + IIc, according to the Paris classification, was present on the anal side of the foramen. Biopsy specimen indicated tubular adenocarcinoma, tub1-2, group 5 (the Japanese Gastric Cancer Classification, 15th edition)

**Fig. 2:**
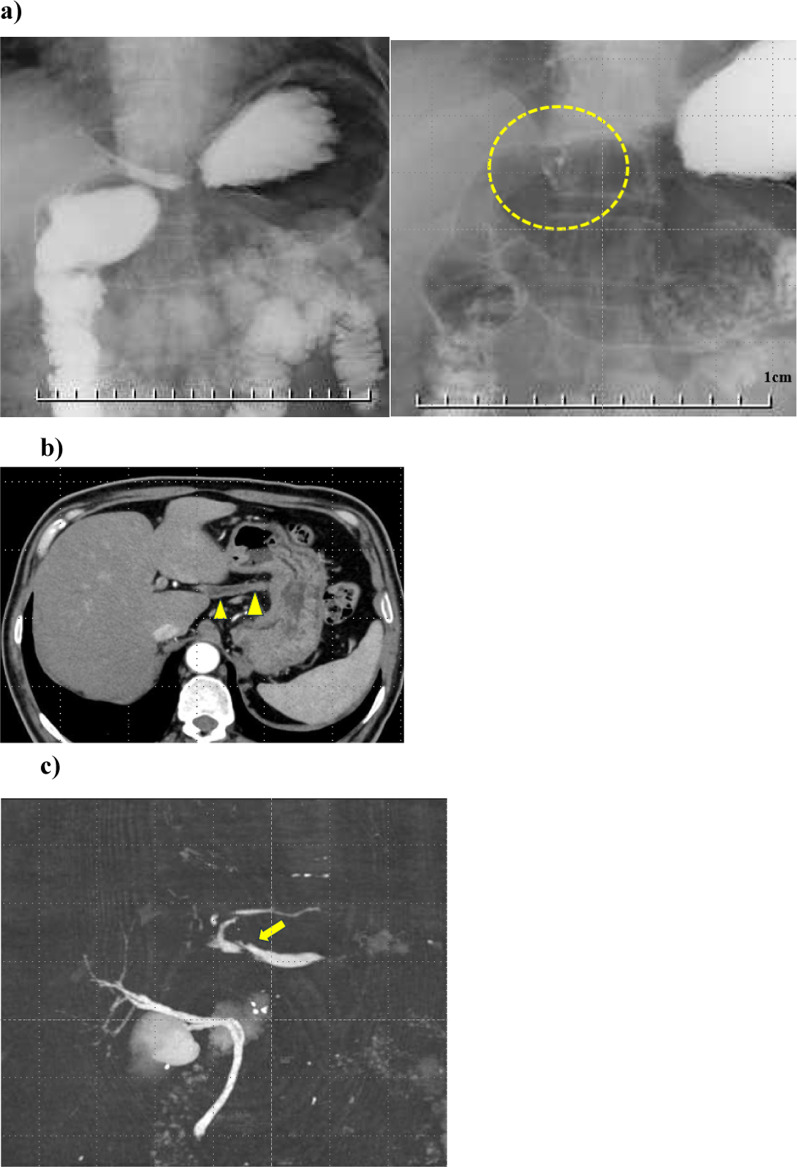
Pre-operative tests. **a** Upper gastrointestinal examination. **b** Computed tomography image showing ectopic bile drainage in the stomach. **c** Magnetic resonance cholangiopancreatography showing a signal defect in the ectopic bile duct and no communication between the left and right intrahepatic bile ducts

A 67-year-old man was referred to our hospital for treatment after being diagnosed with gastric cancer following an upper gastrointestinal endoscopy during a health checkup. Six years prior, the patient was diagnosed with ectopic bile duct drainage in the stomach. After diagnosis of ectopic bile duct, barium fluoroscopy was prohibited; the patient was followed up annually, with upper gastrointestinal endoscopy performed at each follow-up. Upper gastrointestinal endoscopy revealed a bile duct opening in the lesser curvature of the stomach, with bile flowing out of the foramen (Fig. 1a). The tumor was located in the anterior wall of the lesser curvature on the anal side of the foramen, and it was slightly elevated with a central depressed lesion of approximately 25 mm (Fig. 1b). Rough mucosal changes were observed around the pyloric ring. Biopsy of the tumor lesion was performed, leading to the diagnosis of gastric cancer. An upper gastrointestinal examination with a double-contrast study showed a deformity of the gastric angulus and niche in the lesser curvature of the stomach (Fig. 2a). Computed tomography revealed an ectopic bile duct in the lesser omentum with no lymph node swelling or distant metastasis (Fig. 2b). Magnetic resonance cholangiopancreatography revealed ectopic bile duct drainage in the left liver with no communication between the right hepatic ducts. A signal defect was detected inside the ectopic bile duct, suggesting the presence of remnant food or stones (Fig. 2c). Blood tests showed no abnormal findings or evidence of *Helicobacter pylori* infection.

**Fig. 3:**
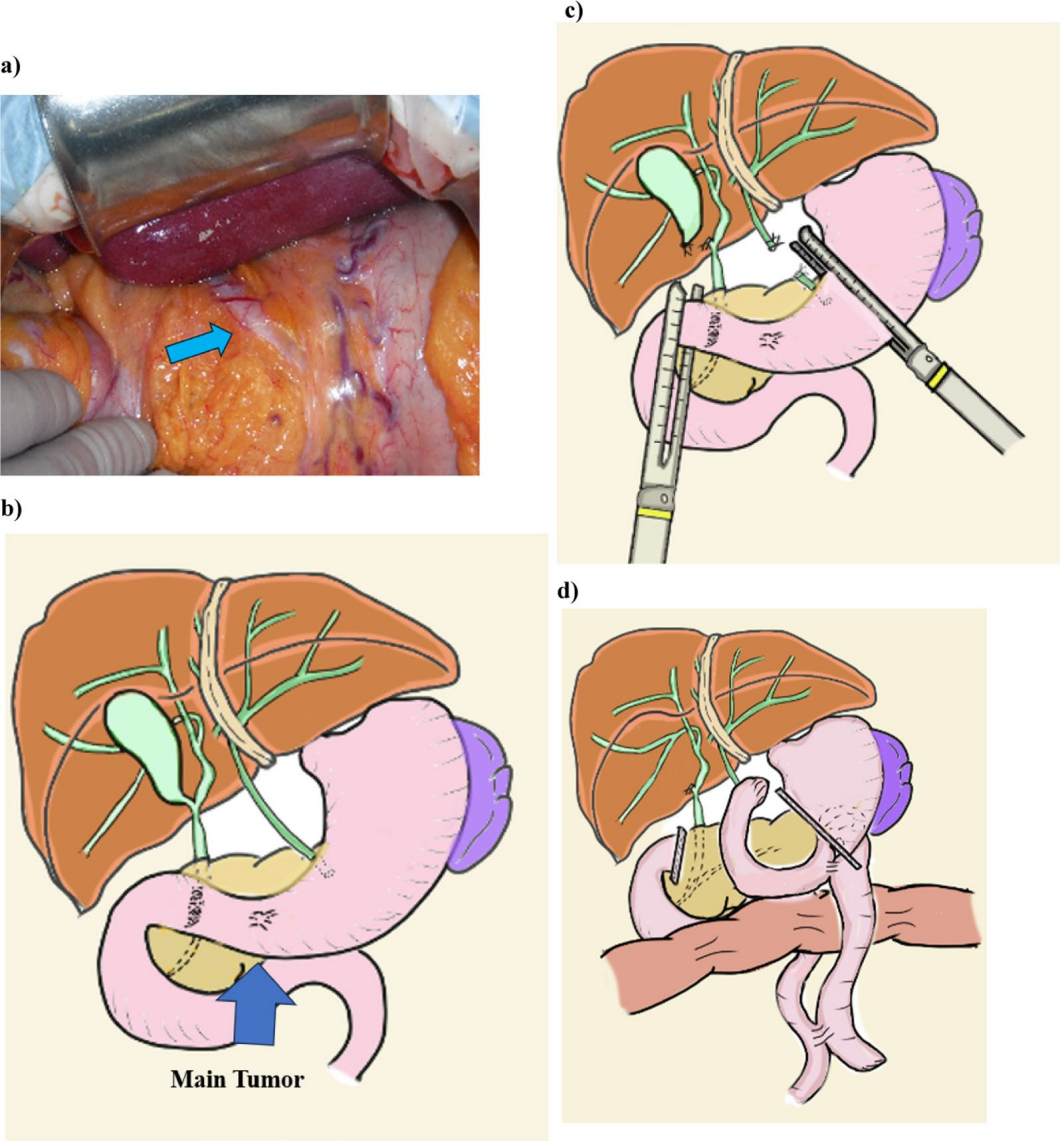
The picture and scheme of the surgical process. **a** Intraoperative photos. The arrow shows the ectopic bile duct running through the lesser omentum and draining into the stomach. **b** Scheme of intraperitoneal findings. **c** An incision was made in the stomach using a surgical stapler and the gallbladder was resected from the liver. The ectopic bile duct was ligated and detached from the stomach. **d** Overview of the procedure after surgery

**Fig. 4:**
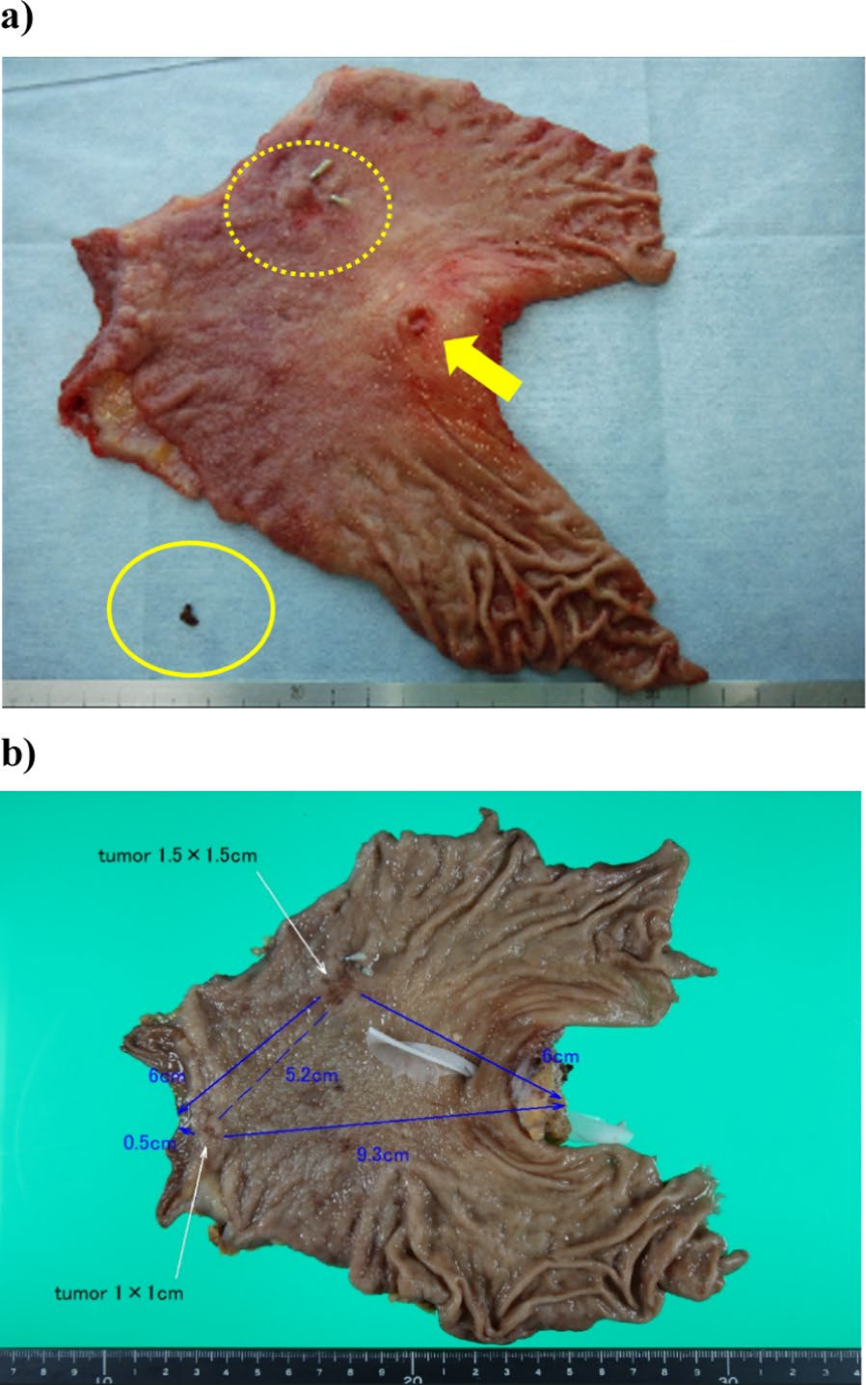
Picture of the resected specimen. **a** The arrow shows the orifice of the ectopic bile duct opening in the lesser curvature of the stomach. The dotted circles show the main tumor lesions. The circle indicates food scraps present in the ectopic bile duct. **b** Arrows indicate tumor lesions. The main tumor (1.5 cm × 1.5 cm) was located 6 cm on the anal side of the oral transection side. A lesion (1.0 cm × 1.0 cm) that could not be diagnosed on preoperative examination was found, located 0.5 cm oral side from the anal transection side

**Fig. 5:**
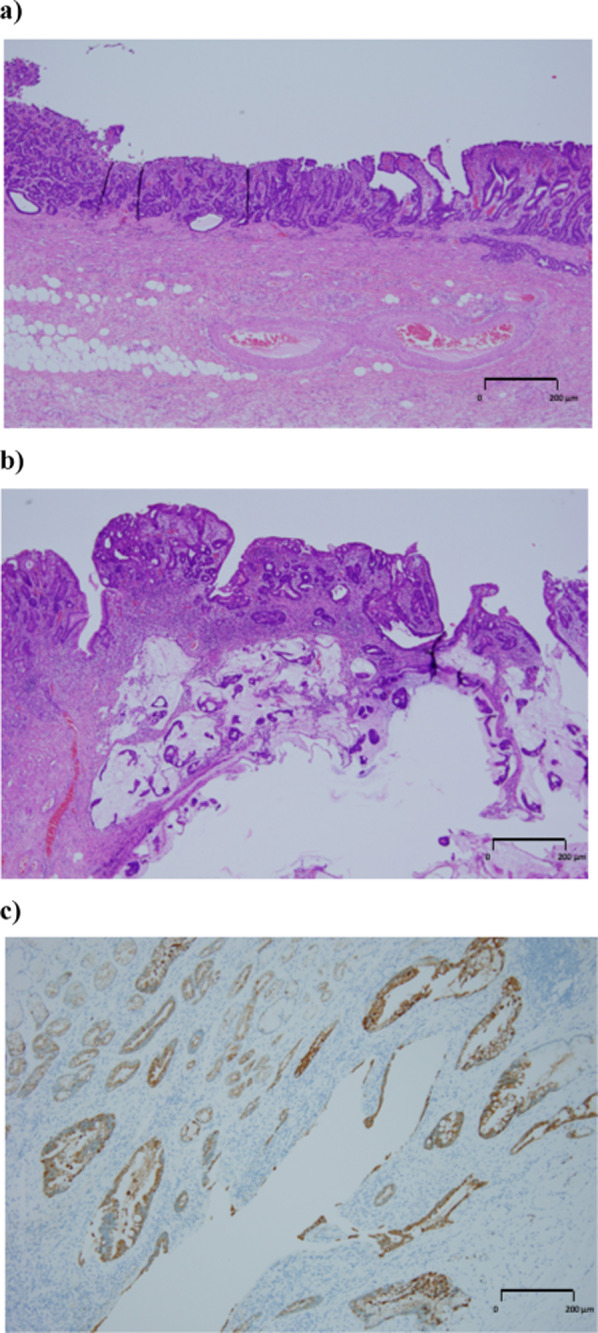
Histopathological view of resected specimens. **a** View of the main tumor. Final pathological findings indicated L, less, type 0–IIc + IIb, 15 × 15 mm, tub1 > tub2, pT1a (M), med, INFb, Ly0, V0, pPM0, pDM0, pN0 (the Japanese Gastric Cancer Classification, 15th edition). **b** View of the tumor lesion around the pyloric ring. Final pathological findings indicated L, less, type 0–IIa, 10 × 10 mm, tub1 > muc > tub2, pT1b (SM), int, INFb, Ly0, V0, pPM0, pDM0, pN0 (the Japanese Gastric Cancer Classification, 15th edition). **c** Immunostaining showing CK19 expression in the muscular layer of the ectopic bile duct. No malignancy was observed in the resected ectopic bile duct

Based on these preoperative findings, we concluded that the clinical stage of the tumor was cT1N0M0 Stage I, according to the Japanese Gastric Cancer Classification, 15th edition. We considered endoscopic submucosal dissection as a treatment option; however, the patient preferred surgical resection because of the possibility of additional surgical resection and simultaneous treatment for ectopic bile duct. Therefore, we performed distal gastrectomy, D1 + lymph node dissection, and gallbladder resection along with gastric jejunostomy (anterior colic), jejunum–jejunum anastomosis, Braun anastomosis, and cholangiojejunostomy for reconstruction (Fig. 3a–d). Food scraps were also present in the ectopic bile. The resected stomach exhibited adequate margins from the tumor. A tumorous lesion was observed in the coarse mucosa around the pyloric ring, which could not be observed on preoperative examination (Fig. 4). Histologically, both lesions were adenocarcinomas localized in the mucosal layer. CK19 expression was observed within the muscular layer of the ectopic bile duct (Fig. 5). The final pathological findings indicated T1b N0 M0 Stage I, according to the Union for International Cancer Control TNM classification.

The patient progressed well without any post-operative complications and was discharged. Post-operative adjuvant chemotherapy was administered for 1 year, and no recurrence was observed until 39 months after the surgery.

## Discussion

Anatomical variations in extrahepatic bile ducts are common, and as many as 47% of patients have been diagnosed with them before surgery. However, a double bile duct with a single drain in the stomach is extremely rare. Kanematsu et al. mentioned that Vesalius first reported a case of double common bile ducts draining into the duodenum and stomach in 1543 [2]. Saito et al. classified double common bile ducts into four main categories (with one classified into two sub-types): (1) Type I, a common bile duct with a septum; (2) Type II, a common bile duct parting on the way and draining individually; (3) Type IIIa, a common bile duct draining independently without separate communication channels (no communication between the left and right bile ducts) and Type IIIb, a common bile duct draining independently with communication channels (ectopic bile duct branching from the right or left hepatic duct to the gastrointestinal tract); and (4) Type IV, a common bile duct draining independently with several separate communication channels [3]. The present case was classified as Type IIIa based on this classification. Therefore, in this case, biliary reconstruction was required instead of just bile duct rejection.

We conducted an electronic literature search in PubMed and Ichushi (in Japanese) to identify cases similar to the present case using the keywords "common bile duct/ ectopic bile duct” and “gastric cancer”. To date, two eligible studies (three cases) have been published, as summarized in Table 1 [4, 5].

**Table 1:** Reported cases of double common bile duct drainage into the stomach in patients with gastric cancer

Author	Article published year	Patient’s age (years)	Sex	Symptoms	Category of common bile duct	Reconstruction of the bile duct
Kondo et al.	1986	37	F	Hunger pain	Not specified	No
		62	M	Hunger pain	Not specified	No
Park et al.	2015	54	M	Indigestion	Type IIIb	No
Present case	2024	67	M	None	Type IIIa	Yes

Bile ducts that drain into organs other than the papilla of Vater can cause several complications. Yamashita et al. reported that 80.1% of all patients with double common bile ducts experienced some symptoms, such as pain around the epigastric and right quadriceps [6]. These symptoms may occur owing to cholangitis and complicated ulcer formation in the distal stomach or bulbar duodenum [7, 8]. In addition to benign diseases, malignant diseases such as gastric and gallbladder cancers are also associated with ectopic common bile ducts [4, 9]. Yamashita et al. reported that 25.5% of ectopic common bile ducts were associated with malignant diseases [6]. In the case of a bile duct with an ectopic drainage into the stomach, persistent bile exposure can lead to carcinogenesis [5]. In the present case, the tumor was located on the anal side of the foramen and was not continuously located near the foramen. In addition, the rough mucosal changes were observed circumscriptively around the pyloric ring containing cancerous components. These findings suggest that gastric cancer occurred accidentally, or that persistent bile exposure and its mixing with refluxed pancreatic juice were the likely causes of gastric cancer in this case. Nevertheless, further studies are required to determine whether sustained bile leaks from ectopic bile ducts can lead to gastric cancer. In cases where this hypothesis is true, we must also consider whether bile diversion surgery or preventive stomach resection is needed in cases of a bile duct with an ectopic drainage into the stomach. In this case, the patient had not undergone surgery but was closely monitored via gastrointestinal endoscopy once a year, leading to early detection.

## Conclusion

Herein, we report a rare case of double common bile duct with ectopic bile duct drainage in the stomach. Although whether sustained bile exposure induces gastric cancer has not been determined conclusively, it is a likely possibility. Therefore, a short-term follow-up is advisable.

## Data Availability

Not applicable.
